# The human disease network in terms of dysfunctional regulatory mechanisms

**DOI:** 10.1186/s13062-015-0088-z

**Published:** 2015-10-08

**Authors:** Jing Yang, Su-Juan Wu, Wen-Tao Dai, Yi-Xue Li, Yuan-Yuan Li

**Affiliations:** School of Biotechnology, East China University of Science and Technology, Shanghai, 200237 P.R. China; Shanghai Center for Bioinformation Technology, 1278 Keyuan Road, Shanghai, 201203 P.R. China; Shanghai Industrial Technology Institute, 1278 Keyuan Road, Shanghai, 201203 P.R. China; Key Laboratory of Systems Biology, Institute of Biochemistry and Cell Biology, Shanghai Institutes for Biological Sciences, Chinese Academy of Sciences, Shanghai, 200031 P.R. China; Shanghai Engineering Research Center of Pharmaceutical Translation, 1278 Keyuan Road, Shanghai, 201203 P.R. China

**Keywords:** Human disease network, Disease similarity, Dysfunctional regulation mechanism, Differential coexpression analysis, Differential regulation analysis

## Abstract

**Background:**

Elucidation of human disease similarities has emerged as an active research area, which is highly relevant to etiology, disease classification, and drug repositioning. In pioneer studies, disease similarity was commonly estimated according to clinical manifestation. Subsequently, scientists started to investigate disease similarity based on gene-phenotype knowledge, which were inevitably biased to well-studied diseases. In recent years, estimating disease similarity according to transcriptomic behavior significantly enhances the probability of finding novel disease relationships, while the currently available studies usually mine expression data through differential expression analysis that has been considered to have little chance of unraveling dysfunctional regulatory relationships, the causal pathogenesis of diseases.

**Methods:**

We developed a computational approach to measure human disease similarity based on expression data. Differential coexpression analysis, instead of differential expression analysis, was employed to calculate differential coexpression level of every gene for each disease, which was then summarized to the pathway level. Disease similarity was eventually calculated as the partial correlation coefficients of pathways’ differential coexpression values between any two diseases. The significance of disease relationships were evaluated by permutation test.

**Results:**

Based on mRNA expression data and a differential coexpression analysis based method, we built a human disease network involving 1326 significant Disease-Disease links among 108 diseases. Compared with disease relationships captured by differential expression analysis based method, our disease links shared known disease genes and drugs more significantly. Some novel disease relationships were discovered, for example, Obesity and cancer, Obesity and Psoriasis, lung adenocarcinoma and *S. pneumonia*, which had been commonly regarded as unrelated to each other, but recently found to share similar molecular mechanisms. Additionally, it was found that both the type of disease and the type of affected tissue influenced the degree of disease similarity. A sub-network including Allergic asthma, Type 2 diabetes and Chronic kidney disease was extracted to demonstrate the exploration of their common pathogenesis.

**Conclusion:**

The present study produces a global view of human diseasome for the first time from the viewpoint of regulation mechanisms, which therefore could provide insightful clues to etiology and pathogenesis, and help to perform drug repositioning and design novel therapeutic interventions.

**Reviewers:**

This article was reviewed by Limsoon Wong, Rui Wang-Sattler, and Andrey Rzhetsky.

**Electronic supplementary material:**

The online version of this article (doi:10.1186/s13062-015-0088-z) contains supplementary material, which is available to authorized users.

## Background

It is increasingly evident that human diseases are not isolated from each other although their clinical and pathological features are diversiform. Understanding how diseases are related to each other can provide novel insights into etiology and pathogenesis [1–4], and furthermore help to prioritize disease-related genes [5–8], perform drug repositioning and drug target identification [9–11].

Early works in this field were limited to examining the overlap in clinical presentation between diseases [4, 11]. For example, Payne *et al*. used logistic regression to estimate the relationships between Alzheimer's disease, Vascular dementia and other types of dementia based on the standardized measures of their clinical impairments, and revealed their notable differences [4]. Four years later, Kalaria *et al*. particularly explored the two extremes of dementia, Alzheimer’s disease and Vascular dementia [11]. These studies improved accurate diagnosis of dementias and aided in clinical decisions on the applicability of different treatments [4, 11]. In 2008, Human Phenotype Ontology (HPO) systematically shows phenotypic similarities of diseases based on clinical synopsis features extracted from OMIM [12].

In recent years, scientists have been able to investigate genetic similarity between diseases based on gene-phenotype relationships [1–3, 8]. Disease relationships were revealed by measuring common disease-related genes or pathways [1, 2], or by clustering both genetic and environmental factors [3]. Moreover, Van Driel *et al*. integrated anatomy information, clinical synopsis, genetic mutation information and medical information context into a feature vector to calculate disease similarities [8]. In some other reports, disease relationships were evaluated by exploring the semantic similarity of disease names or related medical vocabulary concepts according to Disease Ontology (DO) [13, 14] or by checking whether the disease associated enzymes catalyze same/adjacent metabolic reactions or not [15]. Besides, some works combined multi-types of data to identify significant disease relationships [16–18]. It is noted that all these works rely heavily on prior knowledge, therefore they are not applicable when few disease knowledge are available. Also due to the limitation of prior knowledge, it is hard to find out novel disease relationships, or correct ambiguities and errors in the current knowledge repertories.

Fortunately, the rapidly accumulated biomedical data including registry data [15, 18–21] and high-throughput data such as gene expression profiles [9, 10, 16], and the greatly improved data mining strategies offer new chances to discover disease relationships. Based on registry data, scientists can examine the process of disease development by tracing the order of disease occurrence in a large number of patients for a fairly long period of time. In this way, Jensen *et al*. extracted temporal disease trajectories from registry data of 6.2 million Danish patients [19]. Similarly, Blair *et al.* studied the relationships between Mendelian diseases and complex diseases by examining how Mendelian variations enhance the risk of complex diseases according to electronic medical records [20]. Furthermore, Davis *et al.* exploited disease relationships via combining co-morbid diseases in electronic medical records and co-genes diseases in genetic data [18]. These works help to elucidate the process of disease development from a novel viewpoint. However, like the other common big data analysis strategies, these studies can only discover associations, but not causal connections or mechanisms.

In contrast, the genome-scale expression data give us another angle to address this problem since simultaneous measurement of the expression of thousands of genes allows for the exploration of gene transcriptional regulation, which is believed to be crucial to biological functions. In 2009, Hu and Agarwal presented an approach which replaces the pre-existing disease-related genes with differentially expressed genes correlated to diseases, and created a disease-drug network [9]. Similarly, Suthram *et al.* defined the correlation of differential expression values of protein interaction modules between different diseases as the disease similarity measure, and found out 138 significant similarities between diseases [10]. DiseaseConnect, a web server, also utilized differentially expressed genes to explore disease relationships [16]. These studies adopted a common understanding that diseases are highly correlated to the rewiring of gene regulation, which would be manifested at the transcriptional level. However, these dysregulation events are actually difficult to be discovered by traditional differential expression analysis (DEA), while could be captured by differential coexpression analysis (DCEA) [22] since they tend to display as the decoupling of expression correlation. In fact, the DCEA strategy has emerged as a promising method to unveil dysfunctional regulatory mechanisms underlying diseases [22–25]. Following this sense, we propose that a disease similarity measurement based on differential coexpression (DCE), instead of differential expression (DE), may lead to a disease network more relevant to pathogenesis.

In the present work, we developed a DCE-based computational approach to estimate human disease similarity, and identified 1326 significant Disease-Disease links (DDLs for short) among 108 diseases. Benefiting from the use of DCEA, the human disease network is constructed for the first time from the viewpoint of regulation mechanisms.

## Methods

### Gene expression dataset

**Fig. 1:**
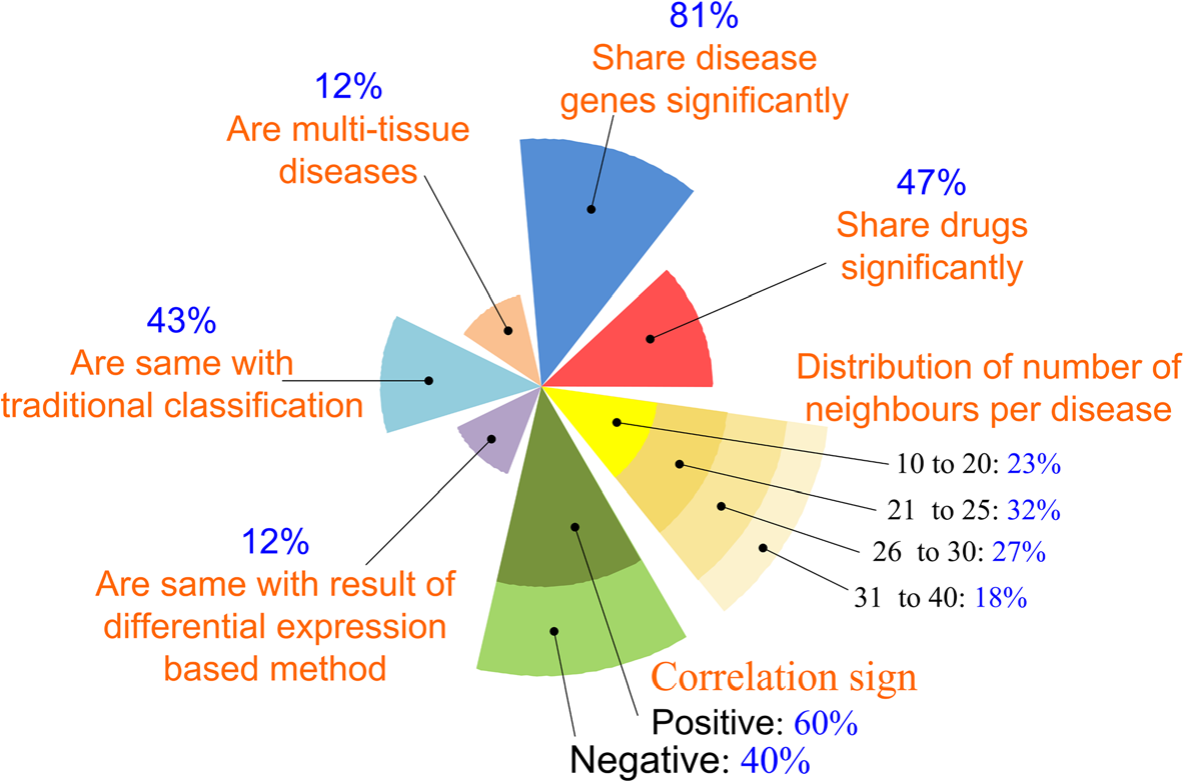
Seven characteristics of disease network. Seven characteristics of our disease network, including its pathogenic relevance (*i.e*., the percentage of disease pairs which significantly share disease genes and drugs), degree distribution (*i.e*., the distribution of the number of disease neighbours), the correlation sign, its comparison with DE-based network, its comparison with tradition disease classification, as well as the percentage of multi-tissue diseases

As of April 19, 2013, we selected 954 GSE datasets (GSE short for GEO series) designed for human studies using Affymetrix U133A chip (*i.e*., GPL96), the most commonly used platform, from GEO (http://www.ncbi.nlm.nih.gov/geo/). We then picked out 106 GSEs which 1) were assigned to human disease condition and corresponding normal condition, 2) had more than five samples in each condition, and 3) came from fresh organs (excluding cell lines). We downloaded raw data (CEL files) of each sample, controlled and removed low quality samples using affy [26] and affyQCReport [27] packages, and finally retained 86 GSE datasets involving 4403 samples for 89 diseases (Additional file 1). In order to carry out a disease-centered analysis, the 86 GSE datasets were re-organized as follows: the datasets which studied the same disease with the same tissue were merged; the datasets which involved multiple diseases or multiple tissues were split. This procedure resulted in 108 datasets, corresponding to 108 diseases (Additional file 1). The disease number was expanded from 89 to 108 because 11 out of the original 89 diseases (12 %, as shown in Fig. 1) involved two or more tissues, which were termed as multi-tissue diseases. A multi-tissue disease was defined by combining its disease name and the originated tissue, for example, Type 2 diabetes - liver and Type 2 diabetes - PBMC (short for peripheral blood mononuclear cell).

### Pathway data

Molecular signature database (MSigDB), a collection of annotated gene sets, includes 7 major collections [28]. A total of 6176 pathways from the following two collections of MSigDB v4.0 were extracted: 1) curated gene sets collected from public pathway databases (such as BIOCARTA. REACTOME, KEGG, etc.), publications in PubMed and knowledge of domain experts, 2) GO gene sets including biological processes, cellular components and molecular functions. In order to reduce the influence of missing data, we excluded pathways whose members were not significantly detected by GPL96 platform by using the binomial probability model. Consequently, we ended up with 5598 pathways covering a total of 21,003 unique genes.

### Disease similarity algorithm

First, we normalized the gene expression data in each microarray sample using MAS5.0 (as shown in Additional file 2, step 1: normalization). Secondly, we calculated the differential coexpression value (dC) of each gene between disease and control samples for all diseases via DCp method which was developed in our previous work [23–25] (as shown in Additional file 2, step 2: calculating genes’ dC). As described in the literatures, DCp was designed for identifying differentially co-expressed genes (DCGs), which proved to be superior to currently popular methods in simulation studies attributed to their uniqueness of exploiting the quantitative coexpression change of each gene pair in the coexpression networks [23–25]. For a certain disease, the Pearson correlation coefficients between gene i and its n neighbors form two vectors, X = (x_i1_, x_i2_, …, x_in_) and Y = (y_i1_, y_i2_, …,y_in_) corresponding to two comparative conditions (say, disease and normal). Finally, dC of each gene for the disease can be calculated with Eq. 1 [23–25].

1 $$ d{C}_i=\sqrt{\frac{{\left({x}_{i1}-{y}_{i1}\right)}^2+{\left({x}_{i2}-{y}_{i2}\right)}^2+\dots +{\left({x}_{in}-{y}_{in}\right)}^2}{n}} $$

Next, similar to what Suthram *et al.* did [10], we assigned dC of pathway to be the average dC of their component genes, and thus obtained a vector of pathways’ dCs for each disease (as shown in Additional file 2, step 3: calculating pathways’ dC). We eventually calculated the partial Spearman correlation coefficient between two diseases as their similarity value (as shown in Additional file 2, step 4: calculating partial correlations). The reason we adopted partial Spearman correlation, instead of generic Spearman correlation, was that partial Spearman correlation was proved to have the capability of factoring out the possible dependencies between different gene-expression experiments due to their underlying tissues [10]. The last step of Additional file 2 for obtaining significant partial correlations will be illustrated in the following section.

### Permutation test of disease pairs

In order to evaluate the statistical significance of observed disease partial correlation coefficients, we randomly re-assigned the affiliation of gene to pathway as Suthram *et al*. did [10]. Pseudo pathways were obtained with the three following values unchanged: 1) the number of pathways a given gene belongs to, 2) the number of pathways’ component genes and 3) the number of all pathways (as shown in Additional file 2, step 5: permutation test), and then calculated the pathways’ dCs and the partial correlation coefficients between every possible disease pairs using the permuted data. This permutation procedure was repeated for 500 times, and the resulting partial correlation coefficient statistics formed an empirical null distribution. In this way, the *p*-value for each disease pair was estimated, and FDR value was obtained accordingly.

### Disease-related genes and drugs

A total of 7357 genes known to be associated with 101 diseases were collected from Genetic Association Database (GAD) [29], Online Mendelian Inheritance in Man (OMIM) [30], Human Gene Mutation Database (HGMD) [31] and human single amino acid variants (SAV) of UniProt (http://www.uniprot.org/docs/humsavar). We also obtained 342 drugs for 83 diseases from DrugBank [32].

### Within-network distance (WD)

According to Li *et al*.’s work, the mean shortest path length among all links in a network was defined as within-network distance (WD) in order to describe the relational closeness of a network (Eq. 2) [2].

2 $$ W{D}_c=\frac{{\displaystyle \sum d\left(i,j\right)}}{k},\kern1.2em i,j\in c $$

Where *k* denotes the total number of links in the network, and *d (i, j)* denotes the shortest path between vertex i and j.

The smaller the WD value, the greater the network compactness. Theoretically when WD = 1, the network is fully connected, displaying as a complete graph.

## Results

### A human disease network was built with a differential coexpression (DCE-) based computational approach

First of all, for each disease, the differential coexpression values (dCs) of all genes were calculated by using differential coexpression algorithm, DCp [23, 24], which was developed in our previous work (see Methods for details). The mean value of the differential coexpression levels of all genes in a certain pathway was calculated as the differential coexpression value of the pathway. In this way, the differential coexpression value (dC) was summarized at the level of biological pathway, which characterized transcriptomic behaviors from a more systematic viewpoint than at the gene level. The disease similarity was then estimated as the partial Spearmen correlation coefficients of pathways’ differential coexpression values (dCs) between any two diseases. Finally, by applying a permutation test, a total of 1326 significant disease relationships at a *p*-value threshold of 0.05 (FDR = 20.91 %), termed as Disease-Disease links (DDLs for short), were identified from all possible links among 108 diseases, leading to a human disease network (see Additional file 3 for details).

According to the basic understanding that similar diseases tend to share similar pathogenesis, and thus have the potential to be treated by common drugs, we assume that the more similarity the diseases display, the more disease-related genes and drugs they share. We therefore checked if the DDLs in our disease network showed this tendency. First, we tidied up a list of known disease genes and a list of drugs (see Methods). The disease genes were found to be associated with 101 out of 108 diseases and 1119 out of 1326 DDLs in our disease network; similarly, the known drugs were correlated to 83 diseases and 745 DDLs. As shown in Table 1, the hypergeometric tests for the 1119-DDL set and 745-DDL set indicated that 910 of 1119 DDLs (81 %, also showed in Fig. 1) significantly shared known disease genes, and 348 of 745 DDLs (47 %, also showed in Fig. 1) significantly shared drugs, both at a *p*-value threshold of 0.05. Among the non-DDL disease pairs, 3732 and 2576 pairs were correlated to known disease genes and drugs, respectively. The hypergeometric tests indicated that 2911 out of 3732 non-DDL pairs (78 %) shared known disease genes significantly, and 1095 out of 2576 (42 %) shared drugs significantly. At last, one-sided Fisher’s exact tests showed that DDLs in our disease network significantly shared both disease-related genes and drugs at a *p*-value threshold of 0.05 (Table 1, 0.009 for disease genes and 0.023 for disease drugs). This verified the reliability of the disease relationships in our disease network at the molecular pathological level.

**Table 1:** Contingency table to validate the assumption that DDLs significantly share disease-related genes or drugs

		Disease pairs correlated to known disease genes or drugs			
		DDLs	Non-DDLs	Total	*P*-value of Fisher’s Exact test
Disease pairs sharing disease genes	Significant	910 (81 %)	2911 (78 %)	3821	0.009085^a^
	Non-significant	209 (19 %)	821 (22 %)	1030	
	Total	1119	3732		
Disease pairs sharing disease drugs	Significant	348 (47 %)	1095 (42 %)	1443	0.02307^a^
	Non-significant	397 (53 %)	1481 (58 %)	1881	
	Total	745	2576		

^a^denotes statistic significance by one-sided Fisher’s Exact test (*p* < 0.05)

Figure 1 summarizes the characteristics of our disease network in terms of its pathogenic relevance (*i.e.,* the percentage of disease pairs which significantly share disease genes and drugs, as listed in Table 1), degree distribution (*i.e.,* distribution of the number of disease neighbours), the correlation sign, its comparison with DE- based network, its comparison with traditional disease classification, as well as the percentage of multi-tissue diseases, which will be explained in details in the following subsections.

With regard to the degree distribution, 60 % diseases in our disease network have 21 ~ 30 neighbour diseases, 23 % have 10 ~ 20 neighbours and 17 % have 31 ~ 40 neighbours, basically following Poisson’s distribution. It is noted that our disease network is a random graph without any hub nodes, contrary to the previous observation by Hu *et al.* [9]. In Hu *et al*.’s work [9], the disease network proved to be a scale-free graph with a few diseases acting as hubs, such as some cancers. We noticed that cancers account for almost half diseases in Hu *et al.*’s network, which is far more than those in our network. Since cancers involve common tumor activators (such as Ras and Myc) and tumor suppressors (such as p53 and PTEN) [33], the rich connections among cancers would make them form hubs. We therefore propose that a disease network which well resembles the heterogeneity of diseasome probably has no hub diseases.

It is interesting that among the 1326 DDLs, 529 (~40 %) links are negative (Fig. 1). When disease A and A’ form a negative link, the patient with disease A tends to be protected from having disease A’ and vice versa, which is probably due to the inversely regulated biological processes involved in the negatively correlated diseases [9]. In agreement with Liu *et al*. opinion, the disease similarity study based on omics data has more chance to find negatively correlated diseases than based on clinical symptom information or gene-phenotype data, because text-mining techniques for clinical symptom information cannot process negative language and gene-phenotype data include disease causal information rather than preventive information [3]. The proportion of negative links in our data (~40 %) is even much higher than Hu *et. al*’s report (~30 %) which adopted differential expression based method to calculate disease similarity [9]. We found that 25 % of Hu *et. al*’s negative links were also sorted out in our data. Since differential coexpression analysis (DCEA) has more potential to discover regulation mechanisms than differential expression analysis (DEA) does, we propose that the negative links which are not included in Hu *et. al*’s work also deserve further investigation. By tracing the differential coexpression properties of a negatively correlated disease pair, one may obtain useful hints for explaining the underlying mechanisms of the mutual exclusion of the two diseases.

### The DCE-based disease network is more relevant to pathogenic mechanisms than the DE-based one

As is mentioned above, differential coexpression analysis (DCEA) is more powerful in unveiling differential regulation mechanisms of diseases than differential expression analysis (DEA) since differential regulations would display as the decoupling of expression correlation [22, 24]. Based on this opinion, we assume that the present differential coexpression (DCE-) based human disease network should be more relevant to pathogenic mechanisms than the networks based on differential expression analysis (DEA).

In order to carry out a parallel comparison, we replaced the dC value in our similarity measurement with differential expression level, the log of Fold Change, and obtained 1583 differential expression (DE-) based DDLs. As expected, one-sided Fisher’s exact tests showed that the DE-based DDLs did not significantly share disease-related genes (*p*-value 0.229) and disease drugs (*p*-value 0.596) (Additional file 4), whose *p*-values were much larger than those of the DCE- based DDLs, 0.009 and 0.023.

**Fig. 2:**
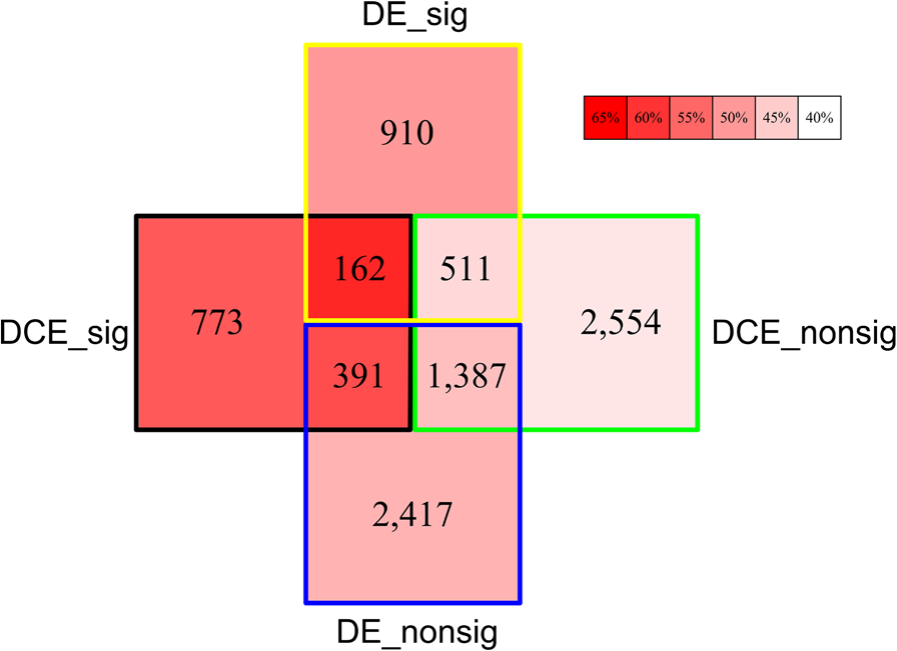
Comparison of two types of disease networks which were identified based on DCE strategy and DE strategy. DCE_sig and DCE_nonsig denote significant and non-significant disease pairs which were identified by differential coexpression based strategy. DE_sig and DE_nonsig denote significant and non-significant disease pairs which were identified by differential expression based strategy. Meanwhile, the depth of color in every region represents the percentages of disease pairs which significantly share disease drugs

To further understand the difference between the two strategies, DE-based and DCE-based, we compared the DDLs identified by the two methods and found that only 162 disease links (~12 %, as shown in Fig. 1) were common. The non-significant disease pairs (DE_nonsig and DCE_nonsig in Fig. 2) were then included in the analysis. According to the percentage of the disease pairs which significantly share drugs (Fig. 2, color depth of each region), it was found that DCE-based DDLs (DCE_sig, including “773” region, “162” region and “391” region in Fig. 2) share drugs much more remarkably than DE-based DDLs (DE_sig in Fig. 2), DE-based non-significant pairs (DE_nonsig in Fig. 2), and DCE-based non-significant pairs (DCE_nonsig in Fig. 2) in order. At the same time, non-significant disease pairs identified by DCE-based method (DCE_nonsig, including “2554” region, “511” region and “1387” region in Fig. 2) have the lowest percentages of the disease pairs which significantly share drugs. However, among the DE-based DDLs (DE_sig, including “910” region, “162” region and “551” region in Fig. 2), the 551 DDLs which are non-significant disease pairs according to DCE-based method have the lowest percentage of the DDLs which significantly share drugs; while, out of the DE-based non-significant pairs (DE_nonsig, including “2417” region, “391” region, “1387” region in Fig. 2), the 391 non-significant pairs which are DDLs according to DCE-based method have the highest percentage of disease pairs sharing drugs. In this way, the disease network based on DCEA proved to be more relevant to pathogenesis than that based on DEA. Figure 2 clearly captures the potential false positive and false negative disease pairs identified by DE-based strategy, and explains why the DCE-based strategy outperformed DE-based strategy.

**Fig. 3:**
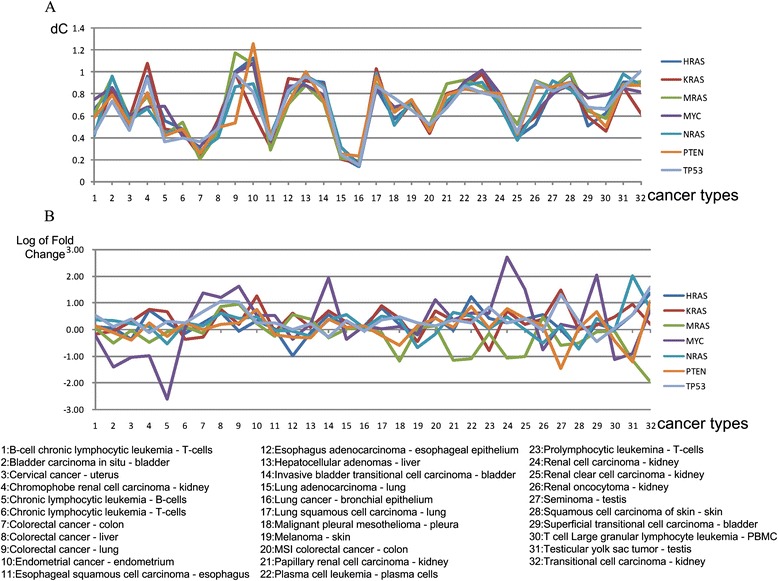
dC values and log values of Fold Change of Ras (NRAS, KRAS, HRAS and MRAS), Myc, p53 and PTEN in 32 cancer datasets. **a** dC values. **b** Log of Fold Change values

In order to compare the relevance of DCE and DE in the present work, we specially extracted 32 cancer datasets from our 108 datasets. Since cancer progression requires the coordination of cancer genes, we simultaneously calculated dC values and the log values of Fold Change of Ras (NRAS, KRAS, HRAS and MRAS), Myc, p53 and PTEN in each cancer type, and proposed that the more relevant the measurement, the more coherent the value across the various cancer genes. It was found that gene dCs in the 32 cancer types are coincident across the seven cancer genes; in contrast, the log of Fold Change didn’t display any significant pattern (Fig. 3 a, b). This result further supports the rationality of our DCE-based analysis strategy.

### The DCE-based human disease network is partly consistent with traditional disease classification

**Fig. 4:**
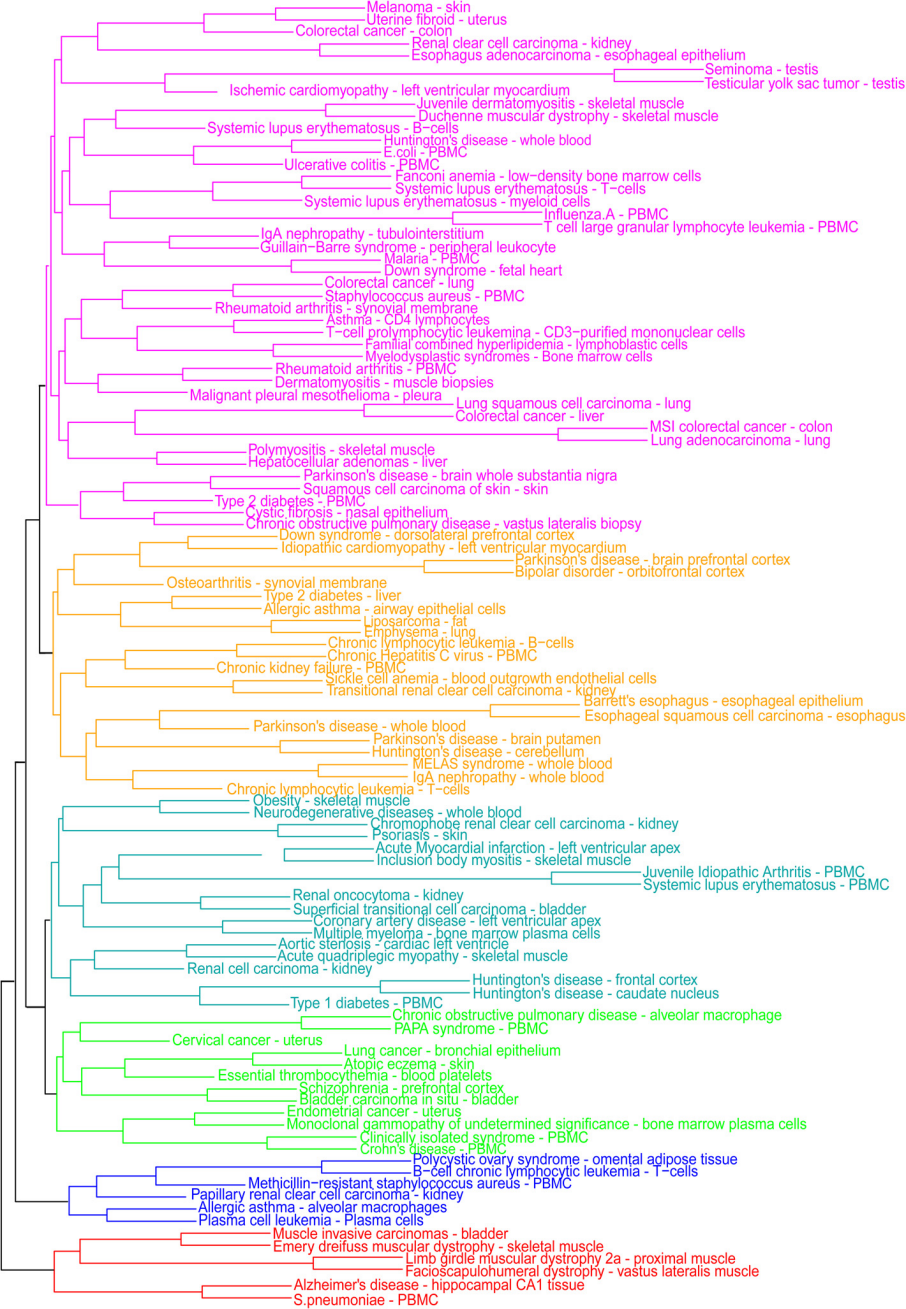
Hierarchical clustering of 108 diseases. Different colors represent different groups

In order to study the consistency of our DCE-based human disease network with previous knowledge on disease classification, we carried out the following analyses. First, we clustered the network by using the average method of hierarchical clustering based on their pair-wise partial correlation coefficients in which non-significant coefficients were set to be zero. This resulted in a cluster tree involving six disease groups, comprised of 6, 6, 12, 18, 22 and 44 diseases respectively (Fig. 4). The six groups are basically consistent with the classification systems in Medical Subject Headings (MeSH), International Classification of Diseases (ICD-10) and Disease Ontology (DO). For example, Neurodegenerative disease, Parkinson’s disease, Alzheimer’s disease and some clinically isolated syndromes which are members of “nervous system disease” category of DO (or “Diseases of the nervous system” category of ICD-10 or “Nervous System Diseases” category of MeSH) are gathered together. Similarly, diseases in “gastrointestinal system disease” category of DO (or “Diseases of the digestive system” category of ICD or “Digestive System Diseases” category of MeSH) such as Ulcerative colitis and Crohn’s Disease are connected.

Furthermore, we marked the 108 diseases in our disease network with their category names in MeSH, ICD-10 and DO, and thus the disease network were divided into several sub-networks according to category markers. In order to check if the diseases from the same category are inclined to form compact sub-network in our disease network, we applied a metric, within-network distance (WD) (see Methods), to estimate the relational closeness of each sub-network [2]. When the WD value of a sub-network is smaller than that of the whole network, the diseases in the sub-network, or within the category, are proposed to lie closer to each other. Table 2 indicates that most of the within-category diseases form more compact sub-networks than the background. Among them, “Infection” and “Mental disorder” are the most compact categories. However, there are a small part of sub-networks/categories which have larger WD scores relative to the whole network, including “disease of anatomical entity” of DO, “Diseases of the musculoskeletal system and connective tissue” of ICD and four categories of MeSH. We checked these categories one by one as follows. Since “disease of anatomical entity” of DO contains several sub-categories, we recalculated the WD scores of the sub-categories with three or more diseases. It was found that all the sub-categories actually show smaller WD scores than the whole network except one, “musculoskeletal system disease” (shown in Additional file 5). As expected, “Diseases of the musculoskeletal system and connective tissue” of ICD and “Skin and Connective Tissue Diseases” of MeSH, which are the congener disease categories of “musculoskeletal system disease” of DO, do not form compact sub-network either. For another three categories of MeSH with larger WD scores than the whole disease network, the scenario is much more complicated at least partly due to the inconsistency of disease taxonomy between DO/ICD-10 and the MeSH system since MeSH allots a disease into multiple categories.

**Table 2:** WD scores for different categories and whole network

	Category names	NO. of diseases	NO. of DDLs	WD scores
DO	disease by infectious agent	7	10	4.20
	disease of anatomical entity^a^	47	248	8.72
	disease of cellular proliferation	38	193	7.28
	disease of mental health	9	10	7.30
	genetic disease	4	2	6.00
ICD-10	Certain infectious and parasitic diseases	5	5	4.00
	Neoplasms	37	179	7.44
	Endocrine, nutritional and metabolic diseases	7	6	7.04
	Disease of nervous system	19	46	7.43
	Disease of the circulatory system	4	2	6.00
	Diseases of the respiratory system	8	8	7.00
	Diseases of the digestive system	3	2	3.00
	Diseases of the musculoskeletal system and connective tissue^a^	11	12	9.17
	Diseases of the genitourinary system	3	2	3.00
MeSH	Neoplasms	36	174	7.24
	Musculoskeletal Diseases	14	12	6.28
	Digestive System Diseases	10	12	7.50
	Respiratory Tract Diseases	8	10	5.60
	Nervous System Diseases	24	72	7.67
	Male Urogenital Diseases	8	10	5.60
	Female Urogenital Diseases and Pregnancy Complications	10	13	6.92
	Cardiovascular Diseases	7	6	7.04
	Hemic and Lymphatic Diseases^a^	11	12	9.23
	Congenital, Hereditary, and Neonatal Diseases and Abnormalities	15	31	6.77
	Skin and Connective Tissue Diseases^a^	12	15	8.80
	Nutritional and Metabolic Diseases	8	9	6.22
	Endocrine System Diseases	4	3	4.00
	Immune System Diseases^a^	23	52	9.73
	Pathological Conditions, Signs and Symptoms^a^	4	1	12.50
	Mental Disorders	7	11	3.84
	Bacteria	3	3	2.00
whole network	1326 DDLs	108	1326	8.71

^a^indicates the categories whose WD scores are larger than that of the whole network

Until now, our disease network prove to be basically compatible with traditional disease classification systems, although some categories have larger WD scores than the whole network, for example, musculoskeletal system disease, which may have more heterogeneity than previously thought and deserve more investigation on their pathogenesis and classification.

At this point, we turned to check the 1326 significant disease relationships (DDLs) in our disease network individually to see if they are consistent with the previous knowledge in MeSH, ICD-10 and DO. It was found that for 566 DDLs (~43 %, see Fig. 1), the disease pair share at least one common disease category. While, the left 760 DDLs (~57 %) are supposed to be novel disease relationships, among which 82.13 % significantly share disease-related genes or drugs (Additional file 3). In fact, some of these novel DDLs have been found to share similar molecular mechanisms by individual studies. For example, in our disease network, Obesity is connected with several types of cancers including Lung squamous cell carcinoma, Lung adenocarcinoma, Colorectal cancer and Renal clear cell carcinoma, which is consistent with several population-based observations [34–36]. In our network, Obesity is also connected with Psoriasis which is located differently from Obesity according to traditional classifications; interestingly, Psoriasis is reported to be affected by many cytokines which contribute to metabolic syndromes such as Obesity [37]. Another novel disease pair, Lung adenocarcima and *S. pneumonia*, is also supported by independent observations, such as the increased risk of lung cancer among persons with lung infections including pneumonia infection [38]. These novel disease relationships will allow more opportunities for drug repositioning, that is, finding new uses of existing drugs.

### Both the type of disease and the type of affected tissue influence disease similarity

Inspired by what the pan-cancer project pointed out, “cancers of disparate organs have many shared features, whereas, conversely, cancers from the same organ are often quite distinct”, we analyzed the similarity of same diseases which originate from different tissues and the similarity of different diseases which originate from the same tissue, aiming to answer if the disease type and the affected tissue influence disease similarity in correlation or not.

**Fig. 5:**
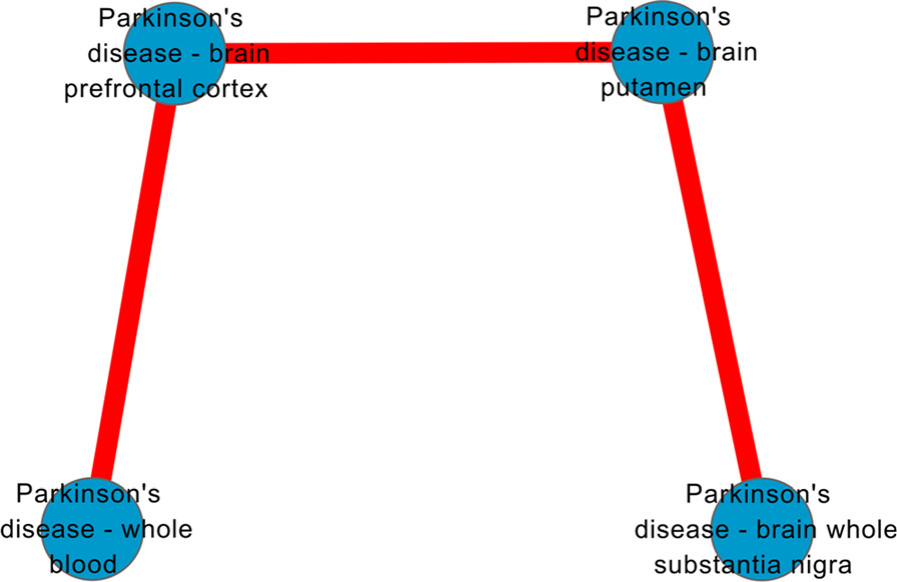
DDLs among four disease members of Parkinson’s disease. Nodes denote Parkinson’s diseases which originated from four tissues, red lines represent positively correlated diseases, and green lines represent negatively correlated diseases

As mentioned above, among the original 89 diseases, 11 diseases involved two or more tissues. For example, Type 2 diabetes split to Type 2 diabetes - liver and Type 2 diabetes - PBMC. We found that the same diseases originating from different tissues (termed as disease members thereafter) could have connections in our disease network, while are not necessarily completely connected. For example, Parkinson's disease - brain whole substantia nigra, Parkinson's disease - whole blood, Parkinson's disease - brain prefrontal cortex and Parkinson's disease - brain putamen are connected as shown in Fig. 5. Another three multi-tissue diseases also show this character, including Chronic obstructive pulmonary disease, Systemic lupus erythematosus and Type 2 diabetes, which have two, four and two disease members respectively. Huntington’s disease is similar: Huntington's disease - frontal cortex is connected with Huntington's disease - cerebellum and Huntington's disease - caudate nucleus, except that Huntington's disease - whole blood is isolated from the other three. For the left six multi-tissue diseases, the disease members are absolutely isolated with each other in our disease network, which include Chronic lymphocytic leukemia, Colorectal cancer, Allergic asthma, Rheumatoid arthritis, IgA nephropathy and Down syndrome involving two, four, two, two, two and two members, respectively. The above observations are consistent with Hoadley’s in cancer [39] that only a small proportion of diseases represent stable manifestations in different tissues. The isolation of disease members in the network indicates that the same diseases could have extremely different pathogeneses in different tissues, just like what the pan-cancer project declared, “same genetic aberrations have very different effects depending on the organ within which they arise” [40].

Furthermore, we systematically estimated if different diseases with same tissue origin tend to show similarity. In the present work, the 108 diseases affect a total of 25 tissues, among which 74 diseases (~69 %, Fig. 1) affect 10 tissues with each tissue involving not less than three diseases. The ten tissues and the related 74 diseases were selected to perform the follow-up analysis. For each tissue, the WD score of the involved diseases was calculated to examine if the different diseases originated from the same tissue form a compact sub-network (Table 3). In order to evaluate the statistical significance of the WD score, we permutated diseases with disease number belonging to a certain tissue unchanged, counted the number of DDLs based on permuted data and calculated the new WD scores. This permutation procedure was repeated 500 times, and the resulting pseudo WD scores formed an empirical null vector. The *p*-value for each tissue was then estimated by using Wilcox test. It was found that WD scores of all the ten tissues are significantly smaller than that of whole disease network except one tissue, lymphoblastic cells. That is, different diseases with same “tissue-of-origin” tend to have similar pathogenesis.

**Table 3:** WD scores for the diseases originated from specific tissues and for the whole network

Tissues	NO. of diseases	NO. of DDLs	WD scores	*P*-values
brain	5	3	1.66	2.346e-06
prefrontal cortex	5	6	2.33	6.29e-06
heart	6	4	2.50	1.351e-10
kidney	7	9	4.00	0.04431
lymphoblastic cells	8	6	22.50	1^b^
lung	6	4	3.00	9.157e-05
muscle	10	15	6.52	0.003319
PBMC^a^	18	43	6.67	7.953e-09
skin	4	3	3.33	0.0176
whole blood	5	7	1.85	9.05e-08
whole network	108	1326	8.71	**-**

^a^PBMC is short for Peripheral Blood Mononuclear Cells

^b^denotes non-significant *p*-value (*p* > 0.05)

Taken together, the traditional disease taxonomy which classifies diseases based on “tissue-of-origin” is reasonable from the viewpoint of disease network in terms of dysfunctional regulatory mechanisms. Additionally, the divergence of disease members in different tissues, the convergence of different diseases in the same tissue, and the stable manifestations in different tissues of a small part of multi-tissue diseases as well, indicate that both the type of disease and the type of affected tissue influence the degree of disease similarity.

### The disease network helps to explore common molecular pathogenesis shared by similar diseases

Since our disease network was inferred by evaluating the similarity of gene correlation change between diseases, it offers us the possibility to explore the common dysfunctional regulation mechanisms underlying DDLs by extracting common differential coexpression relationships shared by linked diseases.

**Fig. 6:**
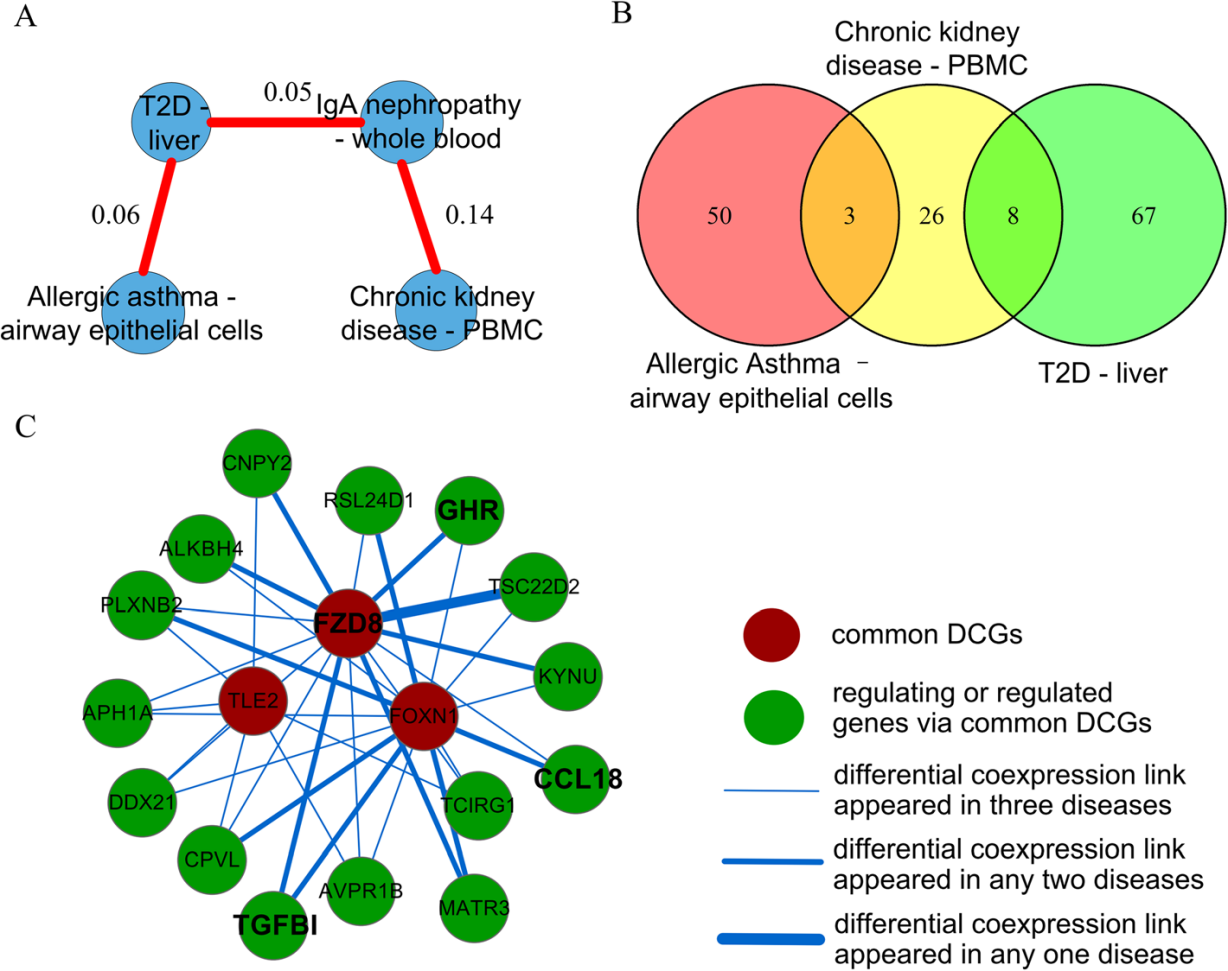
The disease sub-network, disease-related pathways and the differential coexpression information. **a** The sub-network formed by four diseases including Allergic asthma, Type 2 diabetes (T2D), IgA nephropathy and Chronic kidney disease and their partial correlation coefficients. **b** Venn diagram of disease-related pathways of Allergic asthma, T2D and Chronic kidney disease. **c** Gene network centered by three common DCGs in Wnt signaling pathway *FOXN1*, *FZD8* and *TLE2*. Red nodes denote *FOXN1*, *FZD8* and *TLE2*. Green nodes denote genes which form differentially coexpressed links with common DCGs. Four genes with bold type, *FZD8*, *TGFBI*, *CCL18* and *GHR*, denote that their associations with Allergic asthma, T2D or Chronic kidney disease have been reported

We took a sub-network including Allergic asthma, Type 2 diabetes, IgA nephropathy and Chronic kidney disease as an example to demonstrate the exploration of the common pathogenesis underlying the disease network, since the four diseases converged in the same disease cluster in Fig. 4 and were also identified as a disease trajectory which reflects temporal disease progression in Jensen *et al.*’s study [19]. As shown in Fig. 6a, Allergic asthma was connected with Type 2 diabetes, Type 2 diabetes connected with IgA nephropathy, and IgA nephropathy connected with Chronic kidney disease. Considering that IgA nephropathy is linked to several chromosomal regions while the responsible genes are still unclear [41], we excluded IgA nephropathy from the following analysis and focused on the other three relatively better known complex diseases, Allergic asthma, Type 2 diabetes and Chronic kidney disease. We first sorted out 197 common DCGs shared by the three diseases. Meanwhile, we obtained disease-related pathways of the three diseases from MalaCards database version 1.05 [42, 43], resulting in 154 disease-related pathways in total (Additional file 6). However, we noticed that there are no overlapped pathways across the three diseases, and only three pathways are shared between Allergic asthma and Chronic kidney disease, eight shared between Chronic kidney disease and Type 2 Diabetes (Fig. 6b). This is probably attributed to the limited prior knowledge on the disease pathogenesis. We then took the 154 union pathways as candidate pathways of the three diseases. It is interesting that 37 out of 154 pathways (~24 %) are significantly enriched by the 197 common DCGs according to hypergeometric test (see Table 4 for the top three pathways), and the proportion, 24 %, is significantly higher than expected at random by permutation test (*p* = 0.026). Hence, we propose that the 37 pathways and their included 49 common DCGs may contribute to the common molecular pathogenesis of Allergic asthma, Type 2 diabetes and Chronic kidney disease.

**Table 4:** Three the most significant disease-related pathways

Pathways	AA	T2D	CKD	*P*-values	Included common DCGs
WNT_SIGNALING	0.95	0.74	0.60	0.000954	FOXN1, FZD8, TLE2
BIOCARTA_IGF1MTOR_PATHWAY	0.94	0.76	0.61	0.001591	MTOR, IGF1R
KEGG_PROSTATE_CANCER	0.94	0.79	0.62	0.003438	MTOR, IGF1R, IKBKG, E2F2

Numbers in “AA”, “T2D” and “CKD” columns are the differential coexpression values (dCs) of pathways

Among the 37 pathways, Wnt signaling pathway is most significantly enriched by the 197 common DCGs (Table 4). There have been individual reports of associations between Wnt pathway and Asthma [44], Type 2 diabetes [45] and Chronic kidney disease [46], although in MalaCards database Wnt is not assigned to be Asthma or Chronic kidney disease related. According to our data, Wnt pathway involves three common DCGs including *FZD8, FOXN1* and *TLE2*, among which only *FZD8* was reported to participate in the pathogenesis of Asthma [47, 48], and display abnormal expression in Chronic kidney disease [49]. There are no literatures on the roles of *FOXN1* and *TLE2* in Asthma, T2D and Chronic kidney disease in the public domain. We propose that the three genes, *FZD8, FOXN1* and *TLE2* may contribute to the pathogenesis of the three complex diseases. We then identified the differentially coexpressed links (DCLs) by using DCGL [23, 25], and built a gene differential coexpression network which is centered by *FZD8, FOXN1* and *TLE2*, linked by differential coexpression relationships (Fig. 6c). There are a total of 18 genes and 36 links in the network, with 23 links appearing in one disease, 12 links in two diseases, one (the link between *FZD8* and *TSC22D2*) in all three diseases (Additional file 7). According to the philosophy of differential coexpression analysis [24], these links potentially represent the disturbed regulation relationships during disease progression, and therefore are worthy of further investigation.

For example, *FZD8* and *FOXN1* are commonly linked to 14 genes in Fig. 6c, among which *TGFBI* has been proved to contribute to Allergic asthma [47] and T2D [50], *CCL18* contributes to Allergic asthma [51] and *GHR* is associated with T2D [52]. In our data, *TGFBI* and *FOXN1* do not correlate with each other in normal tissue, while they present negative correlation in Allergic asthma (−0.76); meanwhile, the positive correlation of *TGFBI* and *FZD8* in normal tissue is reversed to be negative in T2D (from 0.63 to −0.86). For *CCL18*, it is a differentially coexpressed gene (DCG) in Allergic asthma. As for *GHR*, its negative correlation with *FZD8* in normal tissue (−0.69) disappears in T2D. These correlation changes may indicate altered protein protein interaction, disturbed gene regulation, or some other abnormal molecular events, and therefore provides clues for further investigation of signaling transduction in pathogenesis. It is interesting that none of the above mentioned six genes, *FOXN1*, *FZD8*, *TLE2*, *TFGBI*, *CCL18* and *GHR*, are differentially expressed between disease and normal samples, which is consistent with the opinion that crucial factors are not necessarily differentially expressed [22, 24]. Among the six genes, although *FOXN1*is an immune-related transcription factor (see Additional file 8 for *FOXN1* linked DCLs in the three diseases) and *TLE2* is a transcriptional corepressor that inhibits Wnt signaling, there are not any known regulatory relationships involved in the 36 DCLs in Fig. 6c, which is probably due to the limited number of experimentally validated TFs (199) and their regulation relationships (199,950) in DCGL’s *TF2target* library [25]. With the accumulation of experimental evidences for TFs and their corresponding targets, we believe the present analysis framework could generate more insightful testable hypotheses for pathogenesis studies.

## Discussion

Disease-Disease relationships are of great interest because this knowledge enhances our understanding of disease etiology and pathogenesis. The previous works estimated disease similarities based on commonalities in clinical phenotypes [4, 11], gene-phenotype knowledge bases (OMIM and GAD, for example) [1–3, 8], medical vocabulary concepts/features [13, 14], electronic medical records [15, 18–21], high-throughput data (gene expression profiles, for example) [9, 10, 16] and multi-types of data [16–18]. In this way, disease etiology, pathophysiology and disease-related genes/proteins/microRNAs can be appropriated from one disease to another [5–8]; furthermore, scientists can perform drug repositioning and drug target identification from drug clinical application of similar diseases [9–11]. However, we noticed that when gene expression data were exploited in the field of disease similarities study [9, 10, 16], the attention has only been paid on differential expression. It has been widely accepted that diseases originate from the dysregulation of cell signaling transduction, which causes abnormal expression of a large number of genes. That is, differentially expressed genes are more likely to be the consequences of differential regulation mechanisms, rather than the causes of phenotypic changes. More importantly, a causal factor is not necessarily differentially expressed, for example, when a mutation disrupts the regulation function of the causal factor, the causal factor could still be normally expressed, while in this case, the correlation between the causal factor and its targets will disappear. A new emerging strategy, differential coexpression analysis (DCEA) [22–24], was recently designed to explore gene correlation changes, instead of expression level changes, and has been considered more promising in unveiling differential regulation mechanisms of diseases than differential expression analysis [22]. Therefore, in the present work, we explored the architecture of disease relationships in terms of dysfunctional regulation mechanism by using DCEA for the first time, which has proved to be a complement to the disease networks generated from symptoms, disease concepts, biomedical big data. Benefiting from the use of DCEA, our disease links shared known disease genes and drugs more significantly than disease relationships captured by differential expression (DE) analysis based method (Table 1). By tracing the differentially coexpressed genes and links (DCGs and DCLs), the present disease similarity analysis framework provides a practical way to explore the underlying common molecular mechanisms shared by similar diseases and generate insightful molecular evidences for etiology and pathogenesis.

It is noted that there are quite a lot of novel disease relationships in our disease network, 760 DDLs (~57 % of all DDLs), and most of them (82.13 %) significantly share disease-related genes or drugs (Additional file 3). As mentioned above, the correlations between Obesity and cancer [34–36], Obesity and Psoriasis [37], Lung adenocarcima and *S. pneumonia* [38], and so on, have been reported by pathogenic or epidemiologic studies, although they have not been adopted by traditional disease classification systems. Whereas, by contrast, some diseases that are defined in the same category in traditional classification systems do not show significant similarities in our disease network. It seems that these categories may have more heterogeneity than previously thought and deserve further investigation. We hold the view that a small proportion of diseases need to be reclassified according to new molecular taxonomy. The contradictory observations between our disease network and the traditional disease classification systems may provide insightful clues.

It has been accepted that similar diseases tend to involve similar molecular mechanisms, hence have the potential to be treated by common drugs. That is to say, if a drug has been proved to successfully treat disease A, it might be used to treat A-linked diseases, which is the basis for drug repositioning. Based on the information from DrugBank, the DDLs in our disease network showed this tendency (Table 1). Taking Ulcerative colitis and Crohn’s disease as an example, they are considered as similar diseases in traditional disease classification systems, and their correlation is 0.428 in our data, ranking third among the 1326 DDLs. The *p*-values of the hypergeometric test for their common disease genes and disease drugs are 6.82E-183 and 1.50E-05 respectively, both of which are at top 5 % in all DDLs (Additional file 3). In 1998, Infliximab, a chimeric monoclonal antibody against tumor necrosis factor alpha (TNF-α), was invented and approved for treatment of Crohn’s disease [53]. After several years, some studies proved that Infliximab have positive outcome when treating Ulcerative colitis [54, 55]. We noticed that even among the 760 novel DDLs, 42.7 % significantly share drugs (Additional file 3). For example, Psoriasis and T-cell polymphoytic leukemia are different disease in traditional classification systems, while they form a DDL in our data (correlation coefficient 0.091, at top 8 % in all DDLs). They were found to significantly share drugs with a *p* value of 0.035 (at top 22.7 % in all DDLs, Additional file 3). Methotrexate, an antimetabolite and antifolate drug, is recorded in American Hospital Formulary Service (ASHP) drug information 2004 for treatment of both polymphoytic leukemia and Psoriasis, while methotrexate for autoimmune diseases is taken in lower doses than for cancer [56]. Another interesting example is Parkinson’s disease and Influenza A, which seem to be unrelated with each other; however, Amantadine hydrochloride (trade name Symmetrel, by Endo Pharmaceuticals) has been approved for treatment of both Influenza A and Parkinson’s disease [57]. In our disease network, Parkinson’s disease is linked to Influenza A with the correlation coefficient of 0.061, at top 30 % in all DDLs. They significantly share drugs with *p* value of 0.032 (at top 21 % in all DDLs, Additional file 3).

On the other hand, since negatively correlated diseases, say disease A and A’, may involve inversely regulated biological processes, we proposed that an anti-A drug may have an undesired property of inducing disease A’ when the drug is inversing its target processes. Still taking Crohn’s disease and its therapeutic drug, infliximab, as an example, Crohn’s disease is negatively connected with T-cell source of chronic lymphocytic leukemia (correlation coefficient −0.15, at top 5 %) and Melanoma (correlation coefficient −0.05, at top 50 %) in our data; infliximab, a chimeric monoclonal antibody against tumor necrosis factor alpha (TNF-α), is usually used for treatment of inflammatory bowel disease (IBD) such as Crohn’s disease [53]. In 2006, the Food and Drug Administration (FDA) issued a warning for infliximab given its potential association with the development of Hepatosplenic T-cell lymphoma which is a subtype of T-cell source of chronic lymphocytic leukemia [58]. This phenomenon was also observed in other independent studies [59–61]. Similarly, a case–control study showed an increased risk of melanoma with anti-TNF treatment in IBD patients [62]. We believe that the differential coexpression properties of these negatively correlated diseases could help to explore the underlying mechanisms and improve the therapeutic applications. It is interesting that we also noticed some negatively correlated diseases which shared drug(s). Still taking infliximab as an example, infliximab is used for treatment of both Crohn’s disease [53] and Rheumatoid arthritis [63] although the two diseases formed a negative DDL in our disease network. Another example is tamoxifen, a commonly used anti-breast cancer drug, which was recently proved to remedy Myotonic muscular dystrophy (DMD) in the mdx(5Cv) mouse model [64], though Muscular dystrophy is negatively correlated to some cancers in our disease network. These intriguing observations need further investigation.

We believe that there are valuable druggability information to be discovered in our disease network, and the present work affords an effective and authentic way for systematic drug repositioning. Last but not least, the negative disease pair information helps to discover drug side effects, explore the underlying mechanisms and improve the therapeutic applications.

Just like it was claimed by Todd Golub, “Large, unbiased genomic surveys are taking cancer therapeutics in directions that could never have been predicted by traditional molecular biology [65]”, data-driven disease similarity research strategy allows researchers to get a comprehensive, unbiased architecture of diseasome, which includes useful hints about pathogenesis exploration and drug development.

## Conclusion

We developed a differential coexpression based approach to measure disease similarity, and constructed a human disease network involving 1326 DDLs among 108 diseases. We discovered quite a lot of novel disease links, some of which are being found to share similar pathogenesis. Our data-driven disease similarity strategy allows researchers to obtain a comprehensive, unbiased architecture of diseasome from the viewpoint of dysfunctional regulation mechanisms, which could include hints about pathogenesis exploration and drug development.

## Responses to reviewers

### Reviewers: This article was reviewed by Limsoon Wong, Rui Wang-Sattler and Andrey Rzhetsky

Reviewer's report

Title: The human disease network in terms of dysfunctional regulatory mechanisms

Version: 1 Date: 16 July 2015

Reviewer: Prof Limsoon Wong. School of Computing, National University of Singapore

Report form:

Good points:

1/ This manuscript describes an interesting approach to measure the similarity of diseases by on hypothesized rewiring of gene regulation networks. The rewiring is hypothesized/predicted based on changes in the co-expression of adjacent genes in a pathway. This is an interesting idea and, in theory, is plausible.

2/ The manuscript presents a variety analyses based on the disease-disease network/links generated by the method mentioned in 1/ above. The analyses are interesting and provide reasonable evidence of the validity of the disease-disease links the authors have uncovered. E.g., in one analysis, the enrichment of shared disease genes between adjacent diseases in their inferred disease-disease network is shown.

3/ The manuscript highlights a number of hypotheses on the relationship between diseases; although I am not in a position to judge these, I find them interesting and sufficiently described for a more knowledgeable expert to judge.

4/ I also find the point that if two diseases have a negative relationship, then the drug for one may make the other worse to be interesting and plausible. If this proves valid upon deeper investigation, it points to a very important use of the constructed disease-disease network.

Weak points:

5/ The significance of a disease-disease pair/link is tested by a permutation by random assignment of genes to pathways (albeit preserving number of genes in a pathway, number of pathways, etc.). Nevertheless, such a random assignment is valid only when one assumes as a null hypothesis that genes in a pathway are mutually independent of each other. This null hypothesis is obviously false. Thereby, it has a tendency to be rejected, and this rejection is insufficient for one to conclude the validity of the disease-disease link. The rejection of this null hypothesis (which basically says genes in a pathway are no different from random ones) can only imply an alternate hypothesis that says genes in a pathway do behave differently from a random set of genes. But this alternate hypothesis has nothing to do with the validity of the disease-disease link. I.e., there will be a lot of false positives among the significant links by this permutation test. The authors should think of a more appropriate permutation test (or other form of test) that comes with a more appropriate null hypothesis.

***Response:****We agree with you that our permutation step could be designed more sophisticatedly. We actually borrowed this design from a previously reported DE-based disease similarity study* [10]*. Following its design, we randomized the relationships between genes and pathways while preserving the number of pathways a given gene belongs to, the number of pathways’ component genes, and the number of all pathways. In this way, the distributions of pseudo pathways are similar to their corresponding real pathways, and therefore, we assume that the null hypothesis could be regarded as that the diseases are mutually independent with each other, and the alternate hypothesis is that the diseases links are different from random links.*

*In the present work, we further validated our disease links by checking if the similar diseases in our disease network tend to share disease related genes and drugs (see “A human disease network was built with a differential coexpression (DCE-) based computational approach” section). In order to describe the permutation test more clearly, we revised ‘Permutation test of disease pairs’ in the current version.*

6/ A database of pathways is used as the starting point. It is not clear from the manuscript whether each pathway is used as a separate network and analyzed separately. Or, these pathways are integrated into one single big network, then co-expression analysis is performed on this integrated network. The authors should clarify this in their method description.

***Response:****Sorry, we didn’t make it clear. The coexpression analysis was actually carried out on the gene level at the very beginning of our pipeline, and had nothing to do with pathway knowledge. Since the DCEA method we adopted in the current work has been thoroughly explained in a previous publication, we only cited our original paper and didn’t describe its detailed information. Following the reviewer’s suggestion, we re-organized the method description of “Disease similarity algorithm”) and added a workflow to illustrate the algorithm in the Additional file*2*.*

7/ The manuscript mentions that muscular dystrophy is negatively correlated with cancers. I am not sure that this is consistent with current medical knowledge. For counter examples, I recall myotonic muscular dystrophy has been reported to be associated with elevated risk of cancers and DMD patients have been reported to respond well to cancer drugs like tamoxifen.

***Response:****Thank you for providing this information. We found the paper which reported that tamoxifen, used to treat estrogen-dependent breast cancer, caused remarkable improvements of muscle force and of diaphragm and cardiac structure in the mdx(5Cv) mouse model of myotonic muscular dystrophy (DMD)* [64]*. After rechecking our data, we found another similar example, Crohn’s disease* [53] *and Rheumatoid arthritis* [63]*, which a*re negatively correlated while share a drug, *infliximab. This is quite interesting and deserves further investigation. Since we don’t see any plausible explanations about its potential mechanism, we merely added this observation to the discussion as follows, “It is interesting that we also noticed some negatively correlated diseases which shared drug(s). Still taking infliximab as an example, infliximab is used for treatment of both Crohn’s disease* [53] *and Rheumatoid arthritis* [63] *although the two diseases formed a negative DDL in our disease network. Another example is tamoxifen, a commonly used anti-breast cancer drug, which was recently proved to remedy Myotonic muscular dystrophy (DMD) in the mdx(5Cv) mouse model* [64]*, though Muscular dystrophy is negatively correlated to some cancers in our disease network. These intriguing observations need further investigation.”*

Reviewer's report

Title: The human disease network in terms of dysfunctional regulatory mechanisms

Version: 1 Date: 17 July 2015

Reviewer: Dr Rui Wang-Sattler. Helmholtz Zentrum München, Munich

Report form: see the attached comments

Quality of written English: Acceptable

The manuscript "The human disease network in terms of dysfunctional regulatory mechanisms" presents a human disease network derived from mRNA expression data, pathway data, and information of disease-related genes and drugs. A differential coexpression analysis method, previously developed by the same group, was used to explore the larger data. The authors identified 760 novel disease-disease links and several disease relationships including obesity and cancer. Furthermore, both the types of diseases and of affected tissues were found to influence the degree of disease similarities.

Overall, the design of the study is of high interest. The methods employed are adequate and sound. The analysis is very well done. The results are very promising and provide a good insight into the etiology and pathogenesis. The weaknesses of the paper are the presentation of the complicated results and used methods. The organization of the paper can be improved, for example, a new figure of workflow may help the readers of Biology Direct to better follow and understand the design and results of the study. The clarity and/or coherence of the paper need to be improved as specified as the following:

Specific comments:

Please show words in the ‘Keywords’, e.g., human disease network instead of network.

***Response:****Thanks. “Human disease network” has been shown in the “Keywords”.*

Please remove the description of results from the background section, fourth paragraph, starting with ‘we identified 1326 significant…’.

Please remove the paragraph starting with ‘In conclusion, we construct…’ from the background section, fourth paragraph, as this is also shown in the Abstract.

***Response:****Following your suggestion, we deleted the summary of results and conclusion from the “Background” section. In order to keep the manuscript more complete, we briefly summarized the results at the end of the “Background” section in two sentences, “In the present work, we developed a DCE-based computational approach to estimate human disease similarity, and identified 1326 significant Disease-Disease links (DDLs for short) among 108 diseases. Benefiting from the use of DCEA, the human disease network is constructed for the first time from the viewpoint of regulation mechanisms.”*

The organization of the publication can be improved. The current Results section is partly mixed with methods, introduction and discussion. Please either remove the repeated method description from the results part or move the methods into the Methods section.

For example,

1) In the results, the first paragraph, in general, it’s a description of method starting with ‘As mentioned in the Methods section, a total of 96 ……file 2 for details.’ These should be moved to the method part;

***Response:****Following the reviewer’s suggestion, we removed the repeated method description from the “Results” section, and re-organized the “Methods” section to include all information of data processing. The part of “As mentioned in the Methods section, a total of 96 ……file 2 for details.” was re-wrote and integrated into “Gene expression data” section of “Methods”.*

2) In the results, the third paragraph, starting with ‘In Hu et al.’s work…’ should be introduced in the background;

***Response:****Yes, Hu et al.’s work was introduced in “Background” section. In the third paragraph of “results” section, we focused on the comparison between Hu et al’s work and ours.*

3) In the results, fifth paragraph, please move ‘In 2006, the Food and Drug Administration ….’ to the discussion;

***Response:****Modified. Thanks.*

4) In the discussion, the first three paragraphs till ‘A new emerging strategy…’ can be removed from the manuscript;

***Response:****Modified. Thanks.*

5) In the discussion, the fifth paragraph, some results are first descried in the Discussion section, e.g., ‘…Fig. 6A, B.’, which should be moved to Results section.

***Response:****Modified. Thanks.*

The tables are nicely presented. However, some figures can be improved: Fig. 1A can be removed as 1326 links cannot be seen clearly.

***Response:****We agree with your comment. Have removed the overview diagram of 1326 links from the Figure 1 in the revised version.*

The coloring of Fig. 3 should be different: Once similar diseases and once same tissue should be shown in same colors. Additionally the information should be limited to tissues with more than one available disease and disease groups with more than one tissue measured.

***Response:****We tried to revise Figure 3 according to your suggestion (see the following figure). However, the large number of disease types and tissue types made the graph hard to read. We therefore maintained the original Figure 3.*

For Fig. 4, please explain what is shown and add a legend.

***Response:****Following your suggestion, we added a legend to present the connections among the multi-tissue diseases.*

Please correct these typos:

P10: Please exchange Fig. 1A with 1b, as Fig.1B was first mentioned.

P13: Several abbreviations were defined several times in the method, results and discussion. For example, the differential coexpression analysis (DECA) appeared in numerous places.

Please avoid citing the same reference twice, e.g., Reference no.16 = no. 21.

***Response:****Modified. Thanks.*

Reviewer's report

Title: The human disease network in terms of dysfunctional regulatory mechanisms

Version: 1 Date: 29 July 2015

Reviewer: Andrey Rzhetsky. Institute for Genomics and Systems Biology, University of Chicago

Report form:

The authors’ main assumption is that gene expression in disease is different from“healthy” gene expression in the same tissue type in a partially predictable way. A further assumption is that diseases that share features of expression abnormality for the same tissue type should have partially shared etiology. These assumptions are reasonable and intuitive.

However, there is a disconnect at the point when molecular networks are divided into pathways and one computes the disease similarity statistic over these pathways (“Disease similarity algorithm”): There are numerous ways to split a graph into pathways and the currently-used split was produced using a sequence of somewhat arbitrary decisions. For instance, why is the differential co-expression value best defined by an Euclidean distance between two expression vectors (normalized by a squared root of the number of vector dimensions, equation 1). Are there alternatives? Are there desirable statistical properties or an intuitive physical meaning of a so-defined quantity? In other words, it would be nice if the approach did not depend on the arbitrary decisions of uncoordinated experts.

***Response:****We guess that the order of our descriptions in the “Methods” section seem misleading where the DCp method was explained almost at the last (together with another measure, WD). DCp is actually performed at the very beginning of the pipeline, and it is on the gene level. By using DCp, we obtained the differential coexpression values (dCs) of all genes for every diseases. As pathways are accountable for most processes in the cell, we then calculated the changes in the coexpression levels of various functional pathways of the systems, i.e., dCs of pathways, by calculating the average dC of pathways’ component genes. The disease similarity was finally estimated as the partial Spearmen correlation coefficients of pathways’ dCs between any two diseases. In order to describe the pipeline more clearly, we re-organized “Disease similarity algorithm” section and added a workflow to illustrate each step of the algorithm in Additional file*2*.*

*As for the design of dC measure, since the method was reported in our previous work, we did not explain its details in the current manuscript. As described in the original paper* [24]*, in order to estimate the degree of correlation change of a gene in two contrastive conditions, say disease and normal, the differential coexpression measure, dC, was defined as the Euclidean distance of two contrastive coexpression profile of the gene under two conditions* [24]*. DCp proved to be superior to currently popular designs, including LRC, ASC and WGCNA [Choi, J.K., Yu, U., Yoo, O.J. and Kim, S. (2005) Differential coexpression analysis using microarray data and its application to human cancer. Bioinformatics, 21, 4348–4355. Reverter, A., Ingham, A., Lehnert, S.A., Tan, S.H., Wang, Y., Ratnakumar, A. and Dalrymple, B.P. (2006) Simultaneous identification of differential gene expression and connectivity in inflammation, adipogenesis and cancer. Bioinformatics, 22, 2396–2404. Mason, M.J., Fan, G., Plath, K., Zhou, Q. and Horvath, S. (2009) Signed weighted gene co-expression network analysis of transcriptional regulation in murine embryonic stem cells. BMC Genomics, 10, 327. Fuller, T.F., Ghazalpour, A., Aten, J.E., Drake, T.A., Lusis, A.J. and Horvath, S. (2007) Weighted gene coexpression network analysis strategies applied to mouse weight. Mamm Genome, 18, 463–472. van Nas, A., Guhathakurta, D., Wang, S.S., Yehya, N., Horvath, S., Zhang, B., Ingram-Drake, L., Chaudhuri, G., Schadt, E.E., Drake, T.A. et al. (2009) Elucidating the role of gonadal hormones in sexually dimorphic gene coexpression networks. Endocrinology, 150, 1235–1249.], in simulation studies in retrieving predefined differentially regulated genes and gene pairs, which was attributed to their uniqueness of exploiting the quantitative coexpression change of each gene pair in the coexpression networks.*

I understand that the authors are trying to convince readers that the differential co-expression is biologically more relevant than differential expression. The logic of the comparison of these two types of networks can be made clearer. If I understand correctly, the authors cite their own paper and a commentary piece to claim that it “has been accepted that differential coexpression analysis (DCEA) is more powerful in unveiling regulation mechanisms of disease than differential expression analysis (DEA).” This is, in my view, an overstatement.

***Response:****Due to the space limitation, we did not explain the background of differental coexpression analysis (DCEA) sufficiently in our manuscript. Although DCEA is far from a commonly used method in the field of transcriptomics, differential coexpression and differential regulation have been discussed for more than one decade. Briefly speaking, Differential expression analysis (DEA) looks at absolute changes in gene expression levels, and treats each gene individually. While, gene coexpression analysis explores gene interconnection at the expression level from a systems perspective, and differential coexpression analysis (DCEA) was designed to investigate molecular mechanisms of phenotypic changes through identifying subtle changes in gene expression coordination [Choi, J.K., Yu, U., Yoo, O.J. and Kim, S. (2005) Differential coexpression analysis using microarray data and its application to human cancer. Bioinformatics,****21****, 4348–4355. Reverter, A., Ingham, A., Lehnert, S.A., Tan, S.H., Wang, Y., Ratnakumar, A. and Dalrymple, B.P. (2006) Simultaneous identification of differential gene expression and connectivity in inflammation, adipogenesis and cancer. Bioinformatics,****22****, 2396–2404. Watson, M. (2006) CoXpress: differential co-expression in gene expression data. BMC Bioinformatics,****7****, 509. Fuller, T.F., Ghazalpour, A., Aten, J.E., Drake, T.A., Lusis, A.J. and Horvath, S. (2007) Weighted gene coexpression network analysis strategies applied to mouse weight. Mamm Genome,****18****, 463–472. Mason, M.J., Fan, G., Plath, K., Zhou, Q. and Horvath, S. (2009) Signed weighted gene co-expression network analysis of transcriptional regulation in murine embryonic stem cells. BMC Genomics,****10****, 327. van Nas, A., Guhathakurta, D., Wang, S.S., Yehya, N., Horvath, S., Zhang, B., Ingram-Drake, L., Chaudhuri, G., Schadt, E.E., Drake, T.A. et al. (2009) Elucidating the role of gonadal hormones in sexually dimorphic gene coexpression networks. Endocrinology,****150****, 1235–1249.]. In 2010, a review, entitled “From ‘differential expression’ to ‘differential networking’ – identification of dysfunctional regulatory networks in diseases”, systematically explicated the development from differential expression to differential coexpression for the first time as we know* [22]*. It summarized the purpose and features of differential expression analysis and differential coexpression analysis, and proposed that differential coexpression analysis has more chance to unveil regulation mechanisms of disease than differential expression analysis. Our first paper in this field was published immediately after this review* [24]*. As mentioned above, DCp displays a better performance than its contemporary methods. In very recent years, more and more scientists started to analyze their transcriptomic data from the angle of differential coexpression and differential regulation in order to generate testable hypotheses about the disrupted regulatory relationships or abnormal regulations specific to the phenotype of interest [Diao H, Li X, Hu S, Liu Y (2012) Gene expression profiling combined with bioinformatics analysis identify biomarkers for Parkinson disease. PLoS One 7: e52319. Araki R, Seno S, Takenaka Y, Matsuda H An estimation method for a cellular-state-specific gene regulatory network along tree-structured gene expression profiles. Gene 2013; 518: 17–25. Liu M, Hou X, Zhang P, Hao Y, Yang Y, et al. (2013) Microarray gene expression profiling analysis combined with bioinformatics in multiple sclerosis. Mol Biol Rep 40: 3731–3737. Li G, Han N, Li Z, Lu Q (2013) Identification of transcription regulatory relationships in rheumatoid arthritis and osteoarthritis. Clin Rheumatol. Qu Z, Miao W, Zhang Q, Wang Z, Fu C, et al. (2013) Analysis of crucial molecules involved in herniated discs and degenerative disc disease. Clinics (Sao Paulo) 68: 225–230.]. Following this sense, we proposed that a disease similarity measurement based on differential coexpression (DCE), instead of differential expression (DE), may lead to a disease network more relevant to pathogenesis.*

*In the present work, the disease links in the DCE-based disease network did prove to share known disease genes and drugs more significantly than DE-based disease relationships, supporting that the disease network based on DCEA is more relevant to pathogenesis than that based on DEA. Figure 2 captures the potential false positive and false negative disease pairs identified by DE-based strategy, and explains why the DCE-based strategy outperformed DE-based strategy.*

The whole section comparing DCEA to DEA could be made much clearer by separating assumptions (such as “a good method would have similar diseases share more common drugs”) and results.

***Response:****Thanks. We re-organized the related description, trying to make the assumption and results more readable.*

The partial consistency of the disease classification network with traditional classification is, in my view, not very informative and convincing.

***Response:****Considering that traditional disease classification systems are descriptive conceptual systems, we designed that following analyses to make the comparison. First, we clustered the network by using the average method of hierarchical clustering based on their pair-wise partial correlation coefficients, resulting in a cluster tree including six disease groups (Figure 4). These six groups are basically consistent with the classification systems in Medical Subject Headings (MeSH), International Classification of Diseases (ICD-10) and Disease Ontology (DO). This comparison is similar with previous reports* [9, 10]*. We realized that this so-called consistency is not that informative and convincing, we then applied a metric, WD, to evaluate the consistency as follows, “we marked the 108 diseases in our disease network with their category names in MeSH, ICD-10 and DO, and thus the disease network were divided into several sub-networks according to category markers. In order to check if the diseases from the same category are inclined to form compact sub-network in our disease network, we applied a metric, within-network distance (WD) (see Methods), to estimate the relational closeness of each sub-network* [2]*. When the WD value of a sub-network is smaller than that of the whole network, the diseases in the sub-network, or within the category, are proposed to lie closer to each other. Table 2 indicates that most of the within-category diseases form more compact sub-networks than the background.” Until now, our disease network was proved to be basically compatible with traditional disease classification systems, although some categories have larger WD scores than the whole network.*

Reviewer's report

Title: The human disease network in terms of dysfunctional regulatory mechanisms

Version: 2 Date: 26 August 2015

Reviewer: Prof Limsoon Wong. School of Computing, National University of Singapore

Report form:

The authors did not sufficiently address my earlier comment (#5) that the way the significance of disease-disease pair/link was tested was invalid as the null hypothesis was obviously false. The authors cited an earlier work that used the same strategy as a justification. But would you repeat a mistake when you know it is a mistake just because someone else also make that mistake? I think the authors should make a better effort here. E.g., instead of considering all the randomized pathways they have generated, they should perhaps consider only a subset of their randomized pathways whose genes exhibit a sufficient amount of correlation in their expression level (comparable to correlation levels found among genes in actual pathways of comparable sizes).

***Response:***Following the reviewer’s suggestion, we made a further effort by checking whether our pseudo pathways’ genes exhibit a sufficient amount of correlation or not. First, we calculated the genes’ correlation values of each real pathway in 108 disease expression profiles. That formed a 5598*108 table (please see “/ real_pathway_genes_correlation/ pathway_MoreThanTen_c2c5_0_pathway_multi_exprs.txt” of Additional file9*), which is shortened as the following Table I.*

*Table I. Correlation values of every real pathways in 108 disease expression profiles.*

**Table:** 

*Real pathways*	*Summation of genes’ correlations in Disease 1*	*Summation of genes’ correlations in Disease 2*	*…*	*Summation of genes’ correlations in Disease 108*
*Pathway 1*	*2.5*	*0.6*	*…*	*4.8*
*Pathway 2*	*−1.0*	*−0.8*	*…*	*0.3*
*…*	*…*	*…*	*…*	*…*
*Pathway 5598*	*0.7*	*8.0*	*…*	*10.1*

Then, we calculated the counterpart values for pseudo pathways. For example, based on the 5598 permuted pathways in the first simulation process, we obtained the genes’ correlation values of the pseudo pathways in 108 expression profiles (shortened as Table II). In all, there were a total of 500 5598*108 tables since we permuted the affiliations between genes and pathways for 500 times (due to the limitation of additional file size, please see the result of 1st permutation time at “/pseudo_pathway_genes_correlation/pathway_MoreThanTen_c2c5_1_pathway_multi_exprs.txt” of Additional file9*).*

*Table II. Correlations of 1st permuted pathways in 108 disease expression profiles.*

**Table:** 

*pseudo pathways*	*Summation of genes’ correlations in Disease 1*	*Summation of genes’ correlations in Disease 2*	*…*	*Summation of genes’ correlations in Disease 108*
*Pathway 1*	*2.0*	*−0.8*	*…*	*5.5*
*Pathway 2*	*1.5*	*−2.1*	*…*	*−2.0*
*…*	*…*	*…*	*…*	*…*
*Pathway 5598*	*−2.4*	*−0.4*	*…*	*7.2*

*Also taking the permutation result in Table II as an example, if the correlation value of pseudo pathway n (P*_*n*_*’) in a certain expression profile is within the interval of the real pathway n (P*_*n*_*)’s correlation values in 108 expression profiles, genes of the pseudo pathway (P*_*n*_*’) would be considered as exhibiting a sufficient amount of correlation in the expression profile. Thus, within the 108 expression profiles, we can obtain the proportion of pseudo pathways which present the sufficient amount of correlation. This proportion is termed as sufficient proportion. As shown in Table III, in the 1st permutated process, 80 % (0.80) of correlation values of pathway 1 in 108 expression profiles were within the interval of real pathway 1 in 108 expression profiles.*

*Table III. The sufficient proportions of 108 expression profiles for 5598 pathways in 500 permuted processes.*

**Table:** 

*pseudo pathways*	*1st time*	*2nd time*	*…*	*500th time*
*Pathway 1*	*0.80*	*0.79*	*…*	*0.88*
*Pathway 2*	*0.82*	*0.86*	*…*	*0.92*
*…*	*…*	*…*	*…*	*…*
*Pathway 5598*	*0.89*	*0.85*	*…*	*0.91*
*Distribution of sufficient proportion*	*84 % of 5598 values >0.80*	*83 % of 5598 values >0.80*		*85 % of 5598 values >0.80*

*Finally, we found that almost all sufficient proportions of pseudo pathways in 1st time of permutation are more than 0.5, and the percentage of pseudo pathways in 1st time of permutation whose sufficient proportion values are greater than 0.8 is 84 %. The performances of other 499 permutation times are similar as 1st permutation time (please see “/percentage_of_sufficient_proportion/ percent_sufficient_proportion_of_500_permutation_times.xls” of Additional file*9*). That means most pseudo pathways in our permutation design met the requirement of gene expression correlation, although we did not limit the correlation values of genes when generating pseudo pathways.*

*We really appreciate your careful review and helpful suggestion. All the original calculation results are provided together with this revision.*

The section on exploring common molecular pathogenesis shared by similar diseases is mostly descriptive in nature. It is not clear to me what the real insight is.

***Response:****Since our disease network was inferred by evaluating the similarity of gene correlation change between diseases, it offers us the possibility to explore the common dysfunctional regulation mechanisms underlying DDLs. The section of “The disease network helps to explore common molecular pathogenesis shared by similar diseases” section aims to demonstrate how to explore common molecular pathogenesis shared by similar diseases by extracting common differential coexpression relationships shared by linked diseases, for example, Allergic asthma, Type 2 diabetes, and Chronic kidney disease. In this example, we first sorted out 197 common DCGs shared by the three diseases, and then we integrated disease related pathways to the common DCGs to explore the potential common molecular pathogenesis of the three diseases. Wnt signaling pathway was then extracted, which has been reported to be associated with all the three diseases by individual literatures while has not been recorded in MalaCards database. We therefore highlighted Wnt related DCGs and DCLs, providing clues for those who are interested in the pathogenesis of Allergic asthma, Type 2 diabetes, and Chronic kidney disease. Also, this example demonstrated how to appropriate pathogenesis from one disease to its similar ones in a practical way.*

Also, in the present study, edges are kept if they are significant at *p* <0.05. What if the threshold is changed to *p* < 0.01? Do you observe even stronger evidence for the disease-disease links (e.g., increased in proportion of shared disease genes and drugs)?

***Response:****Following this suggestion, we changed our threshold from p < 0.05 to p < 0.01 and obtained 724 disease pairs (1326 disease pairs when the threshold is p < 0.05).*

*According to the basic understanding that similar diseases tend to share similar pathogenesis, and thus have the potential to be treated by common drugs, we assume that the more similarity the diseases display, the more disease-related genes and drugs they share.*

*As described in the manuscript, when threshold is p < 0.05, a total of 1119 out of 1326 DDLs in the disease network could be associated with known disease genes; similarly, 745 out of 1326 DDLs could be correlated to known drugs (Table 1, Table IV). The hypergeometric tests for the 1119-DDL set and 745-DDL set indicated that 910 of 1119 DDLs (81 %) significantly shared known disease genes, and 348 of 745 DDLs (47 %) significantly shared drugs, both at a p-value threshold of 0.05 (Table 1, Table IV).*

*When threshold is p < 0.01, 599 and 309 out of 724 disease pairs were associated to known disease-related genes and drugs, respectively. Then we applied the same method to evaluate these disease pairs. We found 486 out of 599 (81 %) and 197 out of 309 (64 %) shared disease genes and drugs significantly (Table IV). As the reviewer expected, stronger evidence was observed for the disease links when the p value is changed to 0.01.*

*Table IV. Comparison of disease pairs in different thresholds.*

**Table:** 

		***DDLs when p < 0.05***	***DDLs when p < 0.01***
***Disease pairs sharing disease genes***	*Significant*	*910 (* ***81 %*** *)*	*486 (* ***81 %*** *)*
	*Non-significant*	*209 (19 %)*	*113 (19 %)*
	*Total*	*1119*	*599*
***Disease pairs sharing disease drugs***	*Significant*	*348 (* ***47 %*** *)*	*197 (* ***64 %*** *)*
	*Non-significant*	*397 (53 %)*	*112 (36 %)*
	*Total*	*745*	*309*

## Additional files

Additional file 1:
**Series information of 108 diseases from GEO.** (XLS 41 kb) Additional file 2:
**Workflow for identifying significant Disease-Disease links.** (TIFF 3715 kb) Additional file 3:
**1326 significant Disease-Disease links.** (XLS 358 kb) Additional file 4:
**Contingency table to validate the assumption that DE-based disease relationships significantly share disease-related genes or drugs.** (XLS 21 kb) Additional file 5:
**WD scores for sub-categories of “disease of anatomical entity”.** (XLS 21 kb) Additional file 6:
**154 related pathways in Allergic asthma, Type 2 diabetes and Chronic kidney disease.** (XLS 49 kb) Additional file 7:
***FZD8***, ***FOXN1*** **and** ***TLE2*** **-centered differentially coexpressed links of Allergic asthma, Type 2 diabetes and Chronic kidney disease.** (XLS 34 kb) Additional file 8:
**FOXN1-centered differentially coexpressed links in Allergic asthma, Type 2 diabetes and Chronic kidney disease.** (PDF 747 kb) Additional file 9:
**Results of checking genes’ correlation of pseudo pathways.** (ZIP 9816 kb)

## Abbreviations

DCEA: Differential coexpression analysis
DEA: Differential expression analysis
DCE-based: Differential coexpression based
DE-based: Differential expression based
dC: Differential coexpression value
DCG: Differentially coexpressed gene
DCL: Differentially coexpressed link
DEG: Differentially cexpressed gene
DDL: Disease-Disease link
MeSH: Medical Subject Headings
ICD-10: International Classification of Diseases 10th revision
DO: Disease Ontology
WD: Within-network distance
PBMC: Peripheral blood mononuclear cell
