# Supplementary figures and images for: The human disease network in terms of dysfunctional regulatory mechanisms
Source: Biol Direct. 2015 Oct 8;10:60. doi: 10.1186/s13062-015-0088-z (PMC4599653; doi:10.1186/s13062-015-0088-z)

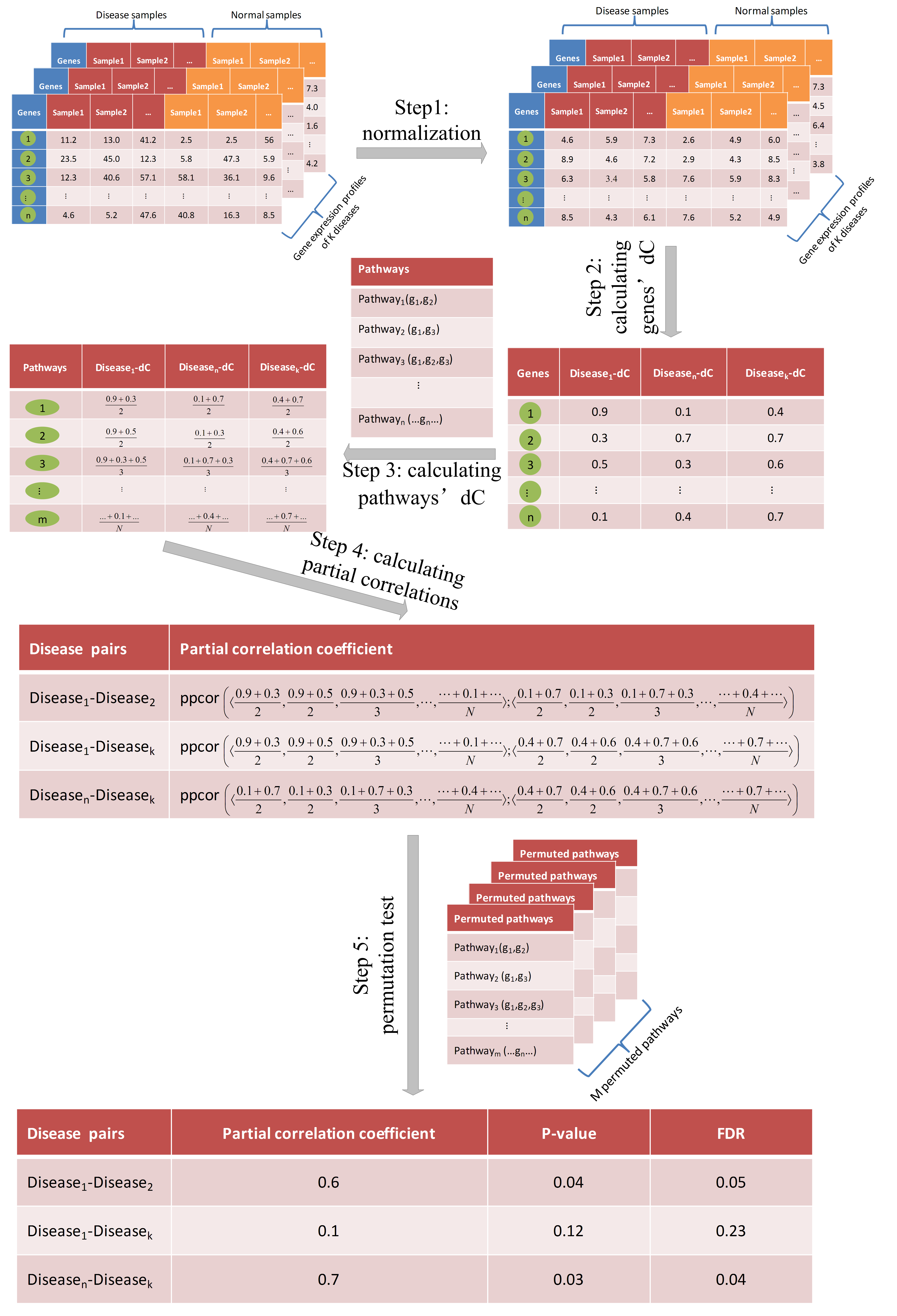

Supplement: Additional file 2: — Workflow for identifying significant Disease-Disease links. (TIFF 3715 kb) [file 13062_2015_88_MOESM2_ESM.tiff]
