# Supplementary material for: The human disease network in terms of dysfunctional regulatory mechanisms
Source: Biol Direct. 2015 Oct 8;10:60. doi: 10.1186/s13062-015-0088-z (PMC4599653; doi:10.1186/s13062-015-0088-z)
Supplement: Additional file 8: — FOXN1-centered differentially coexpressed links in Allergic asthma, Type 2 diabetes and Chronic kidney disease. (PDF 747 kb) [file 13062_2015_88_MOESM8_ESM.pdf]

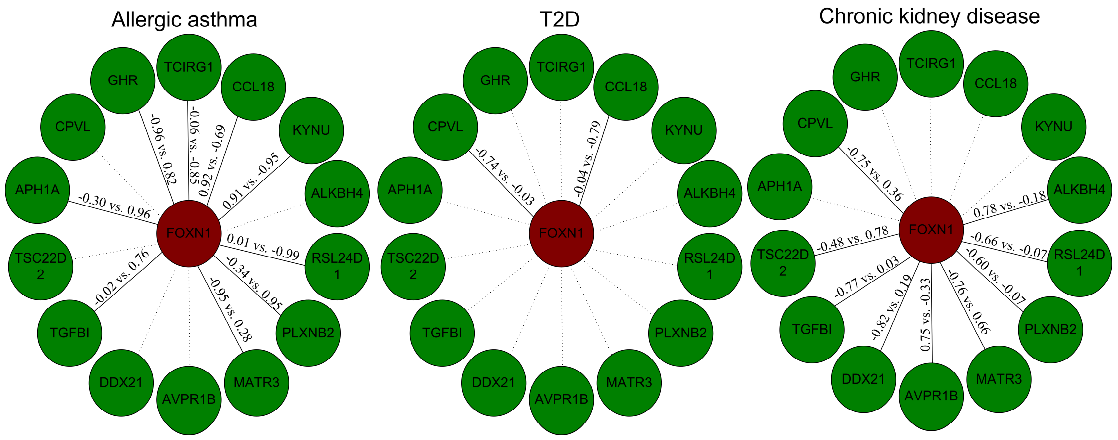

Additional file 8 ***FOXN1*-centered differentially coexpressed links in Allergic asthma, Type 2 diabetes and Chronic kidney disease.** Red nodes denote *FOXN1*, one of three common DCGs. Green nodes denote genes which form differentially coexpressed links with *FOXN1*. Two genes which are linked with dotted line denote the two genes did not form differential coexpressed links in certain disease state. Correlations of each differential coexpressed link in disease samples and accordingly normal samples are showed near the edge (i.e. the correlations of *FOXN1* and *TFGBI* in Allergic asthma samples and accordingly normal samples are written as "-0.02 vs. 0.76").
